# Water and nitrogen regulation strategy for wolfberry farmland based on nitrogen balance in the Yellow River irrigation districts of Gansu Province, China

**DOI:** 10.3389/fpls.2024.1498332

**Published:** 2025-02-03

**Authors:** Minhua Yin, Rongrong Tian, Yi Ling, Yuqing Yang, Yanlin Ma, Yanxia Kang, Guangping Qi, Jinghai Wang

**Affiliations:** ^1^ College of Water Conservancy and Hydropower Engineering, Gansu Agricultural University, Lanzhou, China; ^2^ Key Laboratory of Agriculture Soil and Water Engineering in Arid and Semiarid Areas, Ministry of Education, Northwest A & F University, Xianyang, China

**Keywords:** water and nitrogen regulation, wolfberry, soil NO^-^_3_–N, plant nitrogen uptake, soil N_2_O, nitrogen balance

## Abstract

Agricultural production frequently encounters challenges, including soil nitrogen pollution and imbalances resulting from improper irrigation and fertilization practices. This study focuses on wolfberry farmland, analyzing the effects of four irrigation levels [full irrigation (W0, 75%−85% θ_f_), mild water deficit (W1, 65%−75% θ_f_), moderate water deficit (W2, 55%−65% θ_f_), and severe water deficit (W3, 45%−55% θ_f_)] and four nitrogen application levels [no nitrogen application (N0, 0 kg·ha^−1^), low nitrogen application (N1, 150 kg·ha^−1^), medium nitrogen application (N2, 300 kg·ha^−1^), and high nitrogen application (N3, 450 kg·ha^−1^)] on nitrogen uptake by wolfberry plants, soil nitrogen loss, plant-soil nitrogen balance, and nitrogen use efficiency. The results indicate that: (1) Plant dry matter yield (1338.90−2893.52 kg·ha^−1^), fruit yield (1368.19−2623.09 kg·ha^−1^), plant nitrogen uptake (28.32−96.89 kg·ha^−1^) and fruit nitrogen uptake (23.53−63.56 kg·ha^−1^) all increased with higher irrigation and nitrogen application levels, following the trend W1 > W0 > W2 > W3 and N2 > N3 > N1 > N0. Compared with the other treatments, W1N2 treatment increased by 4.37%−116.11%, 6.36%−91.72%, 15.23%−242.16% and 10.86%−170.13%, respectively. (2) Soil NO_3_^−^–N content initially decreased, then increased, and ultimately decreased again with increasing soil depth, demonstrating inconsistent trends in response to changes in irrigation and nitrogen application. The highest residual soil NO_3_^−^–N at the end of the wolfberry growth period was recorded in the W0N3 treatment, measuring 186.17 kg·ha^−1^. In contrast, the lowest level was observed under the W3N0 treatment at 90.13 kg·ha^−1^, which was reduced by 12.25%−51.59% compared with other treatments. (3) The soil N_2_O flux (28.50–433.41 ug·m^−2^·h^−1^) and total emissions (0.40–1.67 kg·ha^−1^) increased with increased irrigation and nitrogen application. (4) The W1N1 treatment showed the highest nitrogen productivity (14.29 kg·kg^−1^), absorption efficiency (0.85 kg·kg^−1^), and recovery efficiency (27.14%), outperformed other treatments by 0.64–10.94 kg·kg^−1^, 0.10−0.65 kg·kg^−1^, and 2.52–18.80%, respectively. Overall, a combination of 392.40 mm of irrigation and 150 kg·ha^−1^ of nitrogen represented the optimal strategy for efficient and sustainable wolfberry production in the Yellow River irrigation districts of Gansu and similar regions.

## 1 Introduction

Crop growth is a complex and intricate biological process that significantly depends on water and nutrients. Proper allocation of these resources enhances soil fertility and maximizes the potential for agricultural production. In 2022, China’s total water consumption amounted to 599.82 billion cubic meters, with agricultural water usage accounting for 378.13 billion cubic meters (Gao, 2023). However, only 46.3% of the irrigated farmlands use water-saving irrigation techniques. China is the global leader in both fertilizer production and consumption, with an average fertilizer application rate of 328.3 kg·ha^−1^ for crops. This rate is 2.6 times and 2.5 times higher than that of the United States and European Union, respectively (Xie and Zhao, 2022). Wolfberry, recognized for its medicinal properties, role in windbreaks and sand fixation, and ability to enhance saline-alkali soils, is a pioneer species in arid and semi-arid regions that provides both productive and ecological advantages. Currently, China cultivates approximately 100,000 hectares of wolfberry berries, accounting for 80% of the global cultivation area (Gao et al., 2024). However, influenced by the traditional belief that high water and fertilizer inputs lead to high yields, farmers often overuse water and nitrogen resources in wolfberry cultivation, which increases production costs and reduces economic and ecological benefits (Xing et al., 2021). Therefore, optimizing water and nitrogen application strategies in wolfberry cultivation and balancing nitrogen inputs and outputs in farmland systems are crucial for improving resource-use efficiency and reducing the risk of agricultural nitrogen pollution.

Water and nitrogen are key factors that influence crop growth and development and are closely related to the soil nitrogen balance (Chen et al., 2022). Excessive water can result in nitrogen leaching, whereas insufficient water limits nitrogen diffusion and crop uptake (Hu et al., 2024). Adequate water facilitates the dissolution and movement of soil nitrogen, making it more accessible to crop roots. Similarly, when the application of nitrogen fertilizer exceeds crop demand, it not only fails to enhance yield but also reduces nitrogen use efficiency (Zhou et al., 2022). Excessive nitrogen can be lost through runoff, leaching, and volatilization, resulting in environmental issues, such as groundwater nitrate pollution, water eutrophication, and the greenhouse effect (Zhou et al., 2021; Cheng et al., 2019). Research has demonstrated that moderate irrigation and nitrogen application (450 mm, 180 kg·ha^−1^) significantly enhances wheat dry matter yield and nitrogen uptake when compared with high water and nitrogen inputs (600 mm, 225 kg·ha^−1^) (Lv et al., 2020). Additionally, reducing water and nitrogen inputs (60 mm, 150 kg·ha^−1^) reduces the excessive nitrogen by an average of 96.2% and increases nitrogen use efficiency by an average of 95.3% compared with conventional irrigation and nitrogen application (120 mm, 300 kg·ha^−1^), effectively reducing soil NO_3_^−^–N leaching (Guo et al., 2021). A previous study revealed that the highest nitrogen uptake in cotton occurs at an irrigation level of 600 mm and nitrogen application rate of 225 kg·ha^−1^. In contrast, the nitrogen recovery efficiency reaches its peak at the same irrigation level but with a nitrogen application rate of 150 kg·ha^−1^ (Kumar et al., 2022). Compared with traditional irrigation, mild water deficit (81–90% ET), moderate water deficit (69–80% ET), and severe water deficit (54–68% ET) reduce the soil N_2_O emission flux in maize by 50%, 15%, and 40%, respectively (Flynn et al., 2022). Nitrogen application rates ranging from 0 to 187.5 kg·ha^−1^ increase cumulative soil N_2_O emissions in potato cultivation by a factor of 2.3 to 6.7 times compared with the absence of nitrogen application (Zhou et al., 2017).

In summary, current research on the regulation of water and nitrogen in soil-crop systems primarily focuses on grain and cash crops such as wheat, maize, rice, and tomatoes (Chen et al., 2015; Zhang et al., 2020; Tang et al., 2023a; Kou et al., 2021), with limited studies on economically important trees such as wolfberries. The irrigation districts of Gansu Province, situated in the upper reaches of the Yellow River, benefit from abundant sunshine and significant temperature variations between the day and night, making them ideal for wolfberry cultivation. In recent years, the planting area and dried fruit yield of wolfberry in the Yellow River irrigation districts of Gansu Province have accounted for >45% of China’s total, gradually establishing it as a key industry for increasing the income of farmers in the region. However, most of the cultivation of wolfberry in this region adopted flood irrigation combined with about 300 kg·ha^−1^ nitrogen application, which was easy to cause serious soil erosion and salinization. Therefore, the objectives of this study were to (1) analyze the effects of water and nitrogen regulation on wolfberry yield, nitrogen uptake and utilization, and nitrogen balance; (2) quantify nitrogen transport in the plant-soil system under varying water and nitrogen conditions; and (3) develop a water and nitrogen management model for water saving, nitrogen reduction, yield enhancement, and efficiency improvement of wolfberry production. The findings of this study offer valuable insights into the efficient management of water and nitrogen for wolfberry cultivation in the Yellow River irrigation districts of Gansu Province, China, as well as other similar arid regions.

## 2 Materials and methods

### 2.1 Description of the experimental site

**Figure 1:**
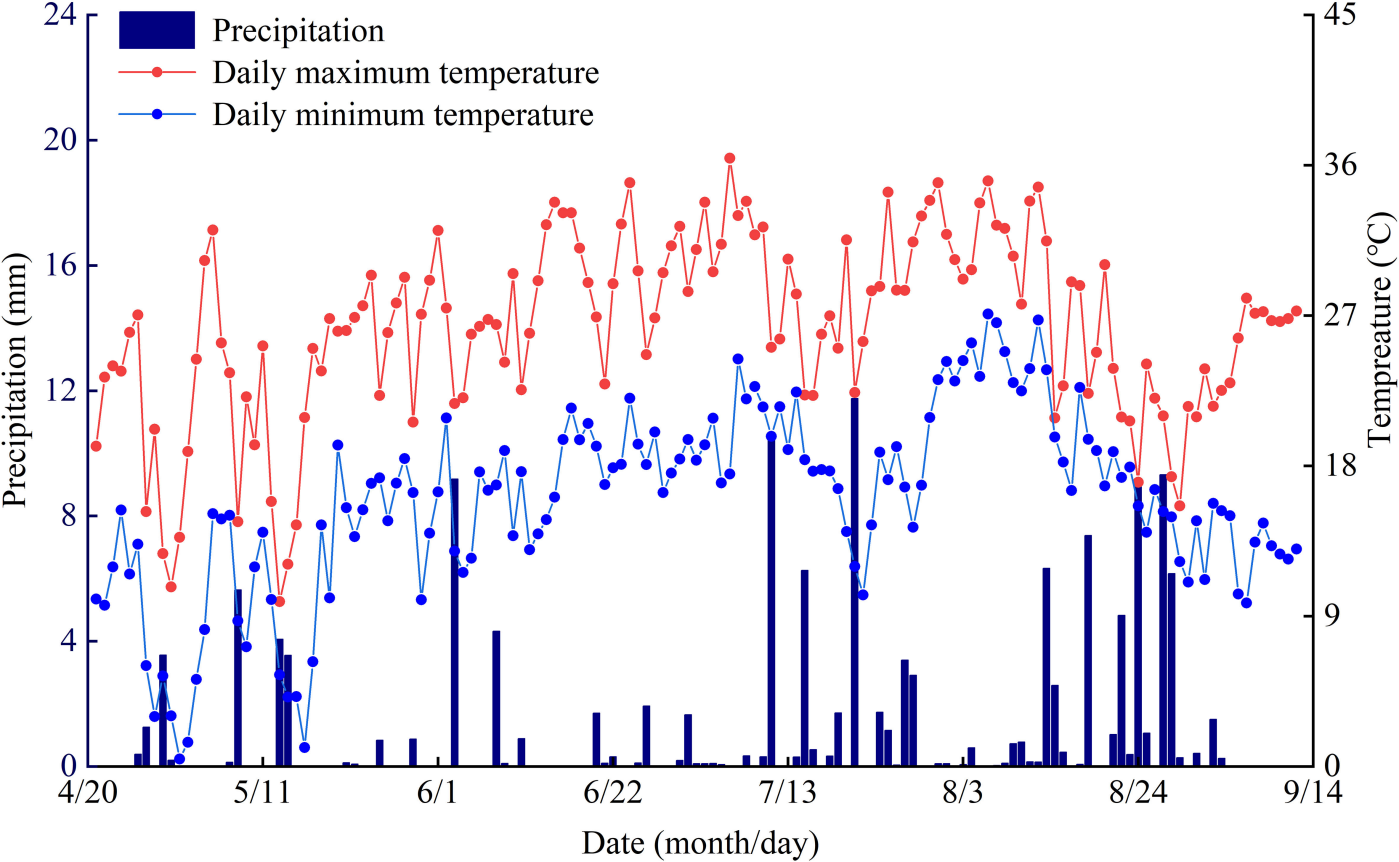
Daily distribution of precipitation and temperature during the experiment.

The experiment was conducted from April to September 2022 at the Jingtaichuan Electric Power Irrigation Water Resource Utilization Center Irrigation Experiment Station in the Gansu Province (37°23′N, 104°08′E). The region has a temperate continental arid climate, characterized by intense sunlight, limited rainfall, and dry conditions. The long-term average values for sunlight duration, frost-free period, solar radiation, temperature, precipitation, and evaporation are 2652 hours, 191 days, 6.18 × 105 J·cm^-^², 8.6°C, 201.6 mm, and 2761 mm, respectively. According to the *Loam Classification Standard* outlined in the *China Soil Classification and Code 2009*, the soil at the experimental site was loamy with a bulk density of 1.63 g·cm^−3^, field capacity of 24.1%, and pH of 8.11. The average contents of total nitrogen, total phosphorus, total potassium, available nitrogen, available phosphorus, available potassium, and alkali-hydrolyzable nitrogen in the 0–60 cm soil layer are 1.62 g·kg^−1^, 1.32 g·kg^−1^, 34.03 g·kg^−1^, 74.51 mg·kg^−1^, 26.31 mg·kg^−1^, 173 mg·kg^−1^, and 55.2 mg·kg^−1^, respectively. Meteorological data were collected using a compact advanced agricultural weather station installed at the experimental site. During the experiment, the total precipitation measured was 137.25 mm, whereas the daily maximum and minimum temperatures were recorded at 36.43°C, and 0.40°C, respectively (**Figure 1**).

### 2.2 Experimental design and field management

The test wolfberry (Ningqi No.5) was a two-year-old seedling transplanted on 12 April 2021, with a plant spacing of 1.5 m and row spacing of 3.0 m. Based on local agricultural practices and previous research findings (Li et al., 2020; Zhao et al., 2021; Su et al., 2019), the experiment used a completely randomized block design with two factors: irrigation and nitrogen application. The irrigation factor controlled the volumetric soil water content as a percentage of the field capacity (θ_f_) during the entire growth period of the wolfberry with a planned wetting depth of 60 cm. This factor included four levels: full irrigation (W0) at 75–85% θ_f_, mild water deficit (W1) at 65–75% θ_f_), moderate water deficit (W2) at 55–65% θ_f_, and severe water deficit (W3) at 45–55% θf. The nitrogen application factor (pure nitrogen) included four levels: no nitrogen (N0, 0 kg·ha^−1^), low nitrogen (N1, 150 kg·ha^−1^), medium nitrogen (N2, 300 kg·ha^−1^), and high nitrogen (N3, 450 kg·ha^−1^). This resulted in a total of 16 treatments (**Table 1**). Each treatment was replicated thrice, resulting in 48 plots, each with an area of 76.5 m² (10.2 m × 7.5 m). Drip irrigation was used and each plot was equipped with an independent valve and a water meter (accuracy: 0.0001 m³) to strictly control the amount of irrigation (**Table 1**). The drip tape was spaced 0.3 meters apart, with a designed emitter flow rate of 2 L·h^−1^ and an emitter spacing of 0.3 meters. Nitrogen fertilizer (urea, containing 46% nitrogen) was applied at a 6:2:2 ratio during the vegetative growth stage (May 21), full flowering stage (June 7), and peak fruiting stage (July 4). Phosphorus fertilizer (superphosphate, containing 12% P_2_O_5_) and potassium fertilizer (potassium chloride, containing 60% K_2_O were each applied at a rate of 130 kg·ha^−1^ as a basal fertilizer during the vegetative growth stage (May 21). Field management and pest control measures were similar to those of local farmers.

**Table 1:** Experimental design.

Treatment	Nitrogen application level (kg·ha^−1^)	Irrigation level (%θ_f_)	
W0N0	0	Full irrigation	75~85
W0N1	150		
W0N2	300		
W0N3	450		
W1N0	0	Slight water deficit	65~75
W1N1	150		
W1N2	300		
W1N3	450		
W2N0	0	Moderate water deficit	55~65
W2N1	150		
W2N2	300		
W2N3	450		
W3N0	0	Severe water deficit	45~55
W3N1	150		
W3N2	300		
W3N3	450		

W0, W1, W2 and W3 refers to full irrigation (75%–85% θ_f_), slight water deficit (65%–75% θ_f_), moderate water deficit (55%–65% θ_f_) and severe water deficit (45%–55% θ_f_), respectively. N0, N1, N2 and N3 refers to the nitrogen application level is 0 kg·ha^−1^, 150 kg·ha^−1^, 300 kg·ha^−1^ and 450 kg·ha^−1^, respectively.

### 2.3 Indicators and methods for measurement

#### 2.3.1 Soil NO_3_^−^–N content (mg·kg^−1^)

**Figure 2:**
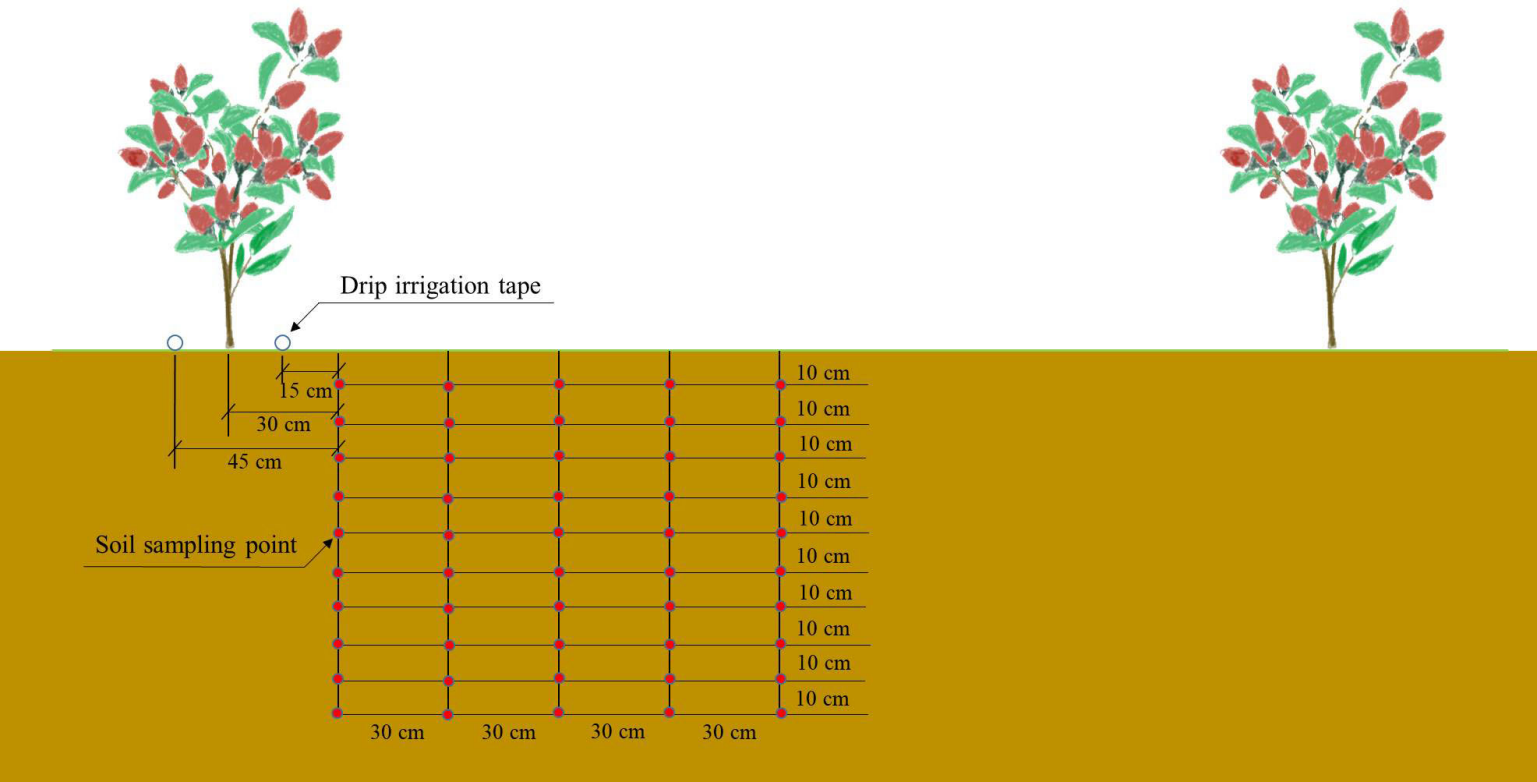
Determination position of NO_3_^−^–N in soil profile.

During the autumn fruiting period of wolfberry (September 5), soil samples were collected using the soil auger method from five positions at distances of 0.3 m, 0.6 m, 0.9 m, 1.2 m, and 1.5 m from the central trunk of the wolfberry plant within each plot. Samples were taken from the 0 to 100 cm soil layer at 10 cm intervals (**Figure 2**). After air-drying, the soil samples were sieved through a 2 mm mesh. The soil NO_3_^−^–N content was extracted using 2 mol·L^−1^ KCl solution at a ratio of 5 g of dry soil to 50 mL of solution (1:10). The extracted NO_3_^−^–N was quantified using a UV-Vis spectrophotometer (T6 New Century, Beijing Purkinje General Instrument Co., Ltd.).

#### 2.3.2 Wolfberry plant dry weight (kg·ha^−1^)

During the autumn fruiting period of the wolfberry (September 8), three representative plants exhibiting typical growth were selected from each plot to sample the roots (using the soil auger method), stems, and leaves. Plant samples were rinsed with distilled water and air-dried. The samples were subsequently divided into roots, stems, and leaves and then dried at 75°C until a constant weight was achieved. The resulting weight was recorded as the dry weight of wolfberry plants.

#### 2.3.3 Total nitrogen content in wolfberry plants (%)

The roots, stems, and leaves collected during the autumn fruiting period of wolfberry were dried, ground, and passed through a 0.5 mm sieve. The samples were subsequently digested using a mixture of H_2_SO_4_ and H_2_O_2_ and the total nitrogen content in each organ was quantified using the Kjeldahl method.

#### 2.3.4 Wolfberry yield (kg·ha^−1^)

From the end of July to the end of August, wolfberries were harvested weekly. The fresh weight of the fruit was measured immediately after each harvest and the total fresh yield for each year was calculated as the sum of the weights from all harvests. Fresh fruits were air-dried to obtain the dried fruits.

#### 2.3.5 Soil N_2_O emissions

During the entire growth period of the wolfberries, soil N_2_O emissions were measured every 3–7 days using closed static chamber gas chromatography (Wu et al., 2022).

### 2.4 Indicator calculation

A nitrogen balance model was established based on the method described by Howarth et al. (1996). Nitrogen inputs to farmland systems include nitrogen from fertilizers, irrigation water, atmospheric nitrogen deposition (both dry and wet), and nitrogen fixation by non-leguminous crops. Nitrogen outputs included leaching of soil NO_3_^−^–N, nitrogen uptake by plants, nitrogen uptake by fruits, and emission of N_2_O from the soil.

#### 2.4.1 Nitrogen inputs in farmland

(1) Nitrogen from irrigation water (*N_I_*, kg·ha^−1^).


(1) $$N_{I}=0.01\times I\times CN_{I}$$

where *I* is the irrigation amount in millimeters and *CN_I_* is the nitrogen concentration in the irrigation water (mg·L^−1^). In this study, the *CN_I_* value was set to 25 mg·L^−1^, following the recommendations of Hong et al. (2010) and Tan et al. (2023) (°C).

(2) Atmospheric nitrogen deposition.

Atmospheric nitrogen deposition included both dry and wet forms. Based on the literature (Liu et al., 2020; Zhang et al., 2021), annual atmospheric nitrogen deposition was set at 74 kg·ha^−1^. Given the duration of the wolfberry growth period of 138 days, atmospheric nitrogen deposition during the growing season was estimated to be 28 kg·ha^−1^.

(3) Nitrogen fixation by non-leguminous crops.

Following Liu et al. (2007) the nitrogen fixation value for non-leguminous crops in this study was set at 20 kg·ha^−1^.

#### 2.4.2 Nitrogen outputs in farmland

(1) Soil NO_3_^−^–N residual (*NR*, kg·ha^−1^) (Cambouris et al., 2008).


(2) NR=γihiNi/10

where $\gamma_{i}$ is the bulk density of the soil of layer *i* (g·cm^−3^), $h_{i}$ is the soil thickness of layer *i* (cm), and $N_{i}$ is the nitrate nitrogen content of the soil in layer *i* (mg·kg^−1^).

(2) Nitrogen uptake by wolfberry plants (*N_u_*, kg·ha^−1^).


(3) $$N_{u}=N_{t}\times W$$

where *N_t_* is the total nitrogen content in the organs of the wolfberry plants (expressed as a percentage) and *W* is the dry weight of the wolfberry plant organs (kg·ha^−1^). The total nitrogen uptake by wolfberry plants is the sum of the nitrogen uptake by each organ.

(3) Nitrogen uptake by wolfberry fruits (*N_uf_*, kg·ha^−1^).


(4) $$N_{uf}=N_{f}\times W$$

where *N_f_* is the total nitrogen content in wolfberry fruit (expressed as a percentage) and *Y* is the wolfberry yield (kg·ha^−1^).

(4) Total N_2_O emissions (*f*, kg·ha^−1^) (Lu et al., 2022).


(5) $$f=\sum [(F_{\text{i}+1}+F_{\text{i}})/2]\times t\times 24/10^{5}$$

where *i* is the number of samples, *t* is the number of days between the *i* sampling time and the *i+1* sampling time (d).

#### 2.4.3 Nitrogen balance in farmland

(1) Soil nitrogen excess or deficit (*N_sp_*, kg·ha^−2^) (Li et al., 2022).

Typically, *N_sp_ >*0 indicates nitrogen excess, *N_sp_* = 0 indicates nitrogen balance, and *N_sp_ <*0 indicates nitrogen deficit.


(6) $$N_{sp}=N_{in}-N_{out}$$

where *N_in_* is the total nitrogen input into the soil (kg·ha^−1^) and *N_out_* is the total nitrogen output from the soil (kg·ha^−1^).

(2) Nitrogen use efficiency level (*N_eul_*, %) (Tai et al., 2021).

This represents the ratio of the total nitrogen output to the total nitrogen input in the soil.


(7) $$N_{eul}=N_{out}/N_{in}\times 100\%$$

(3) Nitrogen input-output ratio (*C_r_*, %).


(8) $$C_{r}=N_{j}/N_{a}\times 100\%$$

where *N_j_* is the amount of a single nitrogen input (or output) (kg·ha^−1^) and *N_a_* is the total nitrogen input (or output) (kg·ha^−1^).

#### 2.4.4 Nitrogen use efficiency

(1) Partial factor productivity of applied nitrogen (*PFPN*, kg·kg^−1^) (Zhang et al., 2018).


(9) PFPN =Y/N

where *N* is the amount of nitrogen applied (kg·ha^−1^).

(2) Nitrogen absorption efficiency (*NAE*, kg·kg^−1^) (Liang et al., 2022).


(10) $$NAE\;=(N_{ur}+N_{us}+N_{ul}+N_{uf})/N$$

where *N_ur_* is the nitrogen uptake by wolfberry roots (kg·ha^−1^), *N_us_* is the nitrogen uptake by wolfberry stems (kg·ha^−1^), *N_ul_* is the nitrogen uptake by wolfberry leaves (kg·ha^−1^), and *N_uf_* is the nitrogen uptake by wolfberry fruits (kg·ha^−1^).

(3) Nitrogen recovery efficiency (*NRE*, kg·kg^−1^) (Ding et al., 2023).


(11) $$NRE\;=(N_{uN}-N_{u0})/N$$

where *N_uN_* is the total nitrogen uptake by wolfberry plants in the N-applied plots (kg·ha^−1^), and *N_u0_* is the total nitrogen uptake by wolfberry plants in the non-nitrogen applied plots (kg·ha^−1^).

### 2.5 Data analysis

The data were statistically analyzed using IBM SPSS Statistics software (version 25.0). One-way analysis of variance (ANOVA) and Duncan’s multiple range tests were performed for variance analysis and multiple comparisons. A two-way ANOVA was used to assess the effects of water and nitrogen, as well as their interactions, on nitrogen inputs and outputs in wolfberry farmland (*P* < 0.05). Figures were created using the Origin 2021 software.

## 3 Results

### 3.1 Nitrogen inputs in wolfberry farmland under varying water and nitrogen regulations

**Figure 3:**
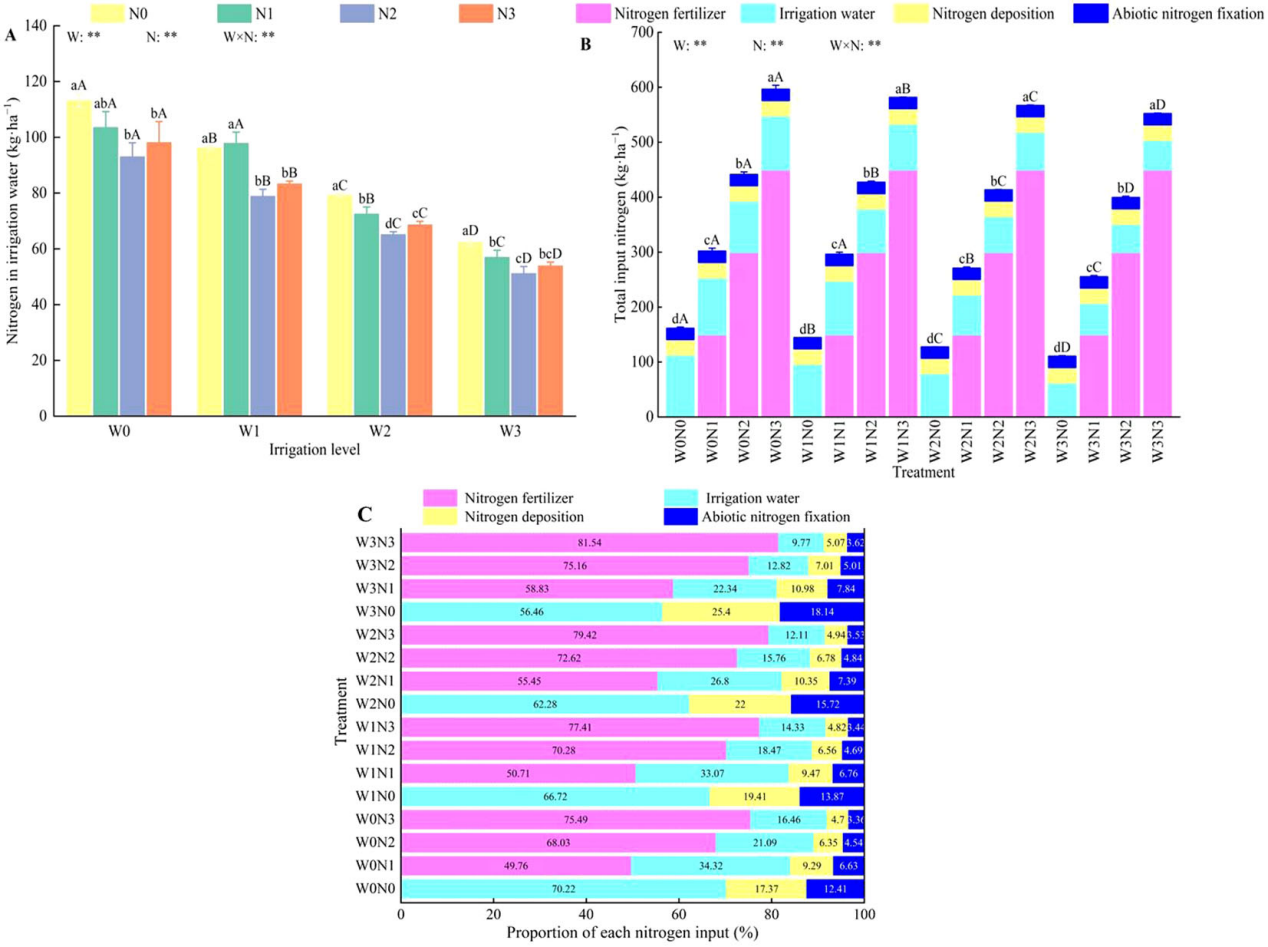
Effect of water and nitrogen regulation on nitrogen input in the field during the growth period of wolfberry. Different lowercase letters indicate the difference between different nitrogen application levels under the same irrigation management, and different capital letters indicate the difference between different irrigation management types under the same nitrogen application level (*P* < 0.05). **(A–C)** represents nitrogen in irrigation water, total input nitrogen and proportion of each nitrogen input, respectively. W and N refer to irrigation and nitrogen application levels, respectively; N × W refers to interaction effect between the two. ** indicates an extremely significant difference (*P* < 0.01). Nitrogen deposition includes dry deposition and wet deposition.

Water, nitrogen and their interactions had highly significant effects (*P* < 0.01) on the nitrogen content of irrigation water (**Figure 3A**) and total nitrogen input (**Figure 3B**). At the same irrigation level (except W1), the nitrogen content in the irrigation water initially decreased and subsequently increased as the nitrogen application rate increased. The N2 treatment resulted in average increases of 17.88%, 12.90%, and 5.20% compared with N0, N1, and N3, respectively. At the same nitrogen application level, the nitrogen content of the irrigation water increased with the volume of irrigation applied. Specifically, W3 exhibited average increases of 45.01%, 37.03%, and 21.43% compared with W0, W1, and W2, respectively. Among all the treatments, the W0N0 treatment exhibited the highest nitrogen content from irrigation water (113.21 kg·ha^−1^) (**Figure 3A**).

The total nitrogen input in farmland ranged from 110.27–596.15 kg·ha^−1^, with nitrogen fertilizer being the primary source, followed by nitrogen from irrigation water, atmospheric nitrogen deposition (dry + wet), and nitrogen fixation by non-leguminous crops (**Figure 3B**). These sources contributed 49.76%–81.54%, 9.77%–70.22%, 4.70%–25.40%, and 3.36%–18.14% of the total nitrogen input, respectively (**Figure 3C**). The W3N0 treatment exhibited the lowest total nitrogen input at 110.27 kg·ha^−1^, which was between 2.49% and 81.50% lower than that of the other treatments.

### 3.2 Nitrogen outputs in wolfberry farmland under varying water and nitrogen regulations

#### 3.2.1 Nitrogen uptake by wolfberry plants

(1) Plant dry matter and fruit yield.

**Figure 4:**
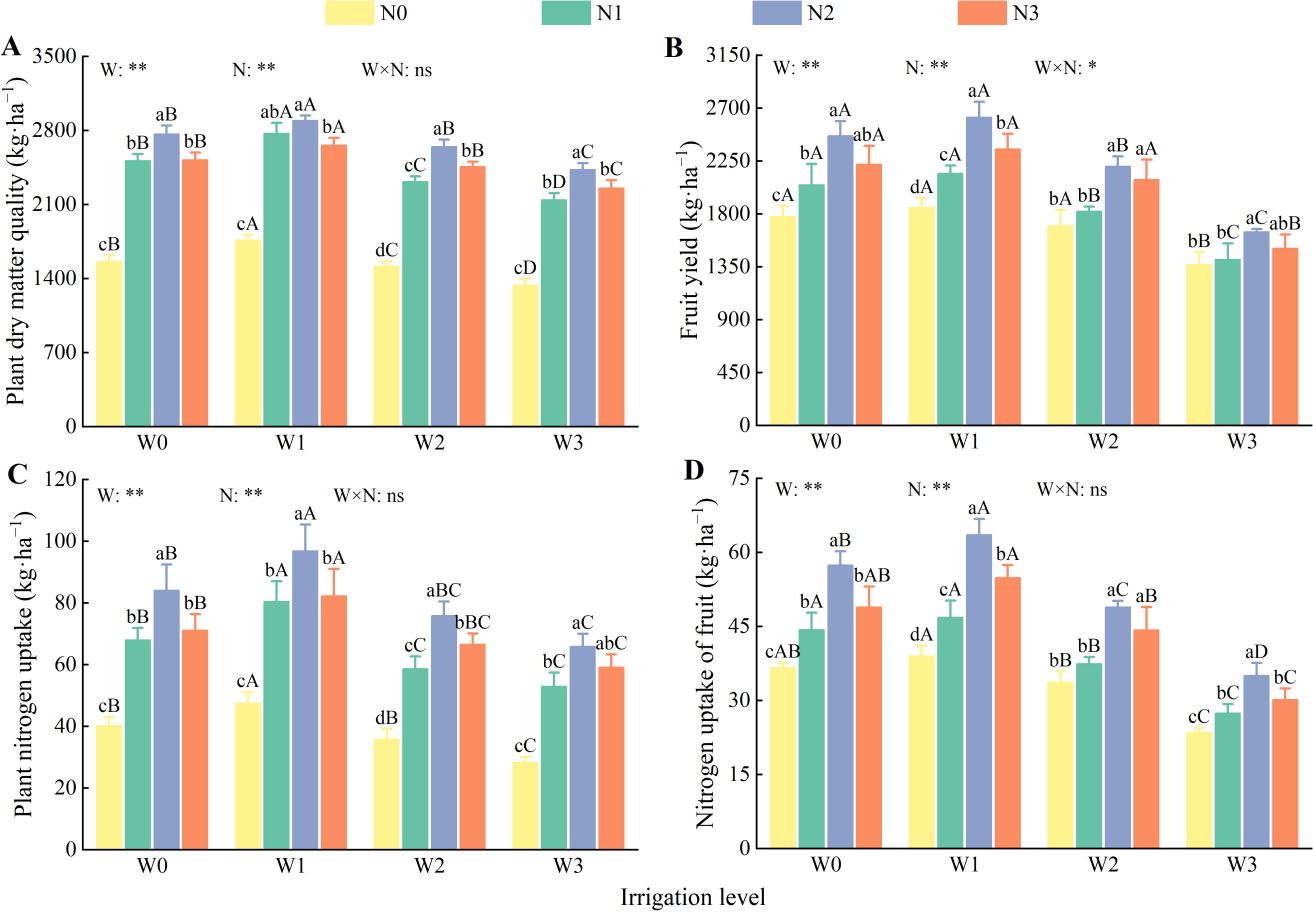
Effects of water and nitrogen regulation on dry matter quality, yield and nitrogen uptake of wolfberry. Different lowercase letters indicate the difference between different nitrogen application levels under the same irrigation management, and different capital letters indicate the difference between different irrigation management types under the same nitrogen application level (*P* < 0.05). **(A–D)** represents plant dry matter quality, fruit yield, plant nitrogen uptake and nitrogen uptake of fruit, respectively. W and N refer to irrigation and nitrogen application levels, respectively; N × W refers to interaction effect between the two. ** indicates an extremely significant difference (*P* < 0.01); * indicates a significant difference (*P* < 0.05); ns indicates no significant difference (*P* > 0.05).

Water, nitrogen, and their interactions significantly affected the dry matter and fruit yield of the wolfberry plants (*P* < 0.05, **Figures 4A, B**). At the same irrigation level, the dry matter and fruit yield first increased and subsequently decreased with increasing nitrogen application, reaching their peak at the N2 level. Compared with N0, N1, and N3, the dry matter and fruit yield at N2 were significantly higher by 64.10%–81.57%, 4.37%–14.25%, 7.65%–9.66%, and 20.38%–41.37%, 16.67%–22.36%, 5.42%–11.48%, respectively. At the same nitrogen application level, both dry matter and fruit yield first increased and subsequently decreased with increasing irrigation, reaching their peaks at the W1 level. Compared with W0, W2, and W3, the dry matter and fruit yield at W1 were significantly higher by 4.57%–12.89%, 8.30%–19.70%, 18.07%–31.69%, and 4.41%–6.36%, 9.23%–18.97%, 35.67%–59.26%, respectively. Among all treatments, W1N2 produced the highest plant dry matter (2893.52 kg·ha^−1^) and fruit yield (2623.09 kg·ha^−1^).

(2) Nitrogen uptake by plants and fruits.

Irrigation and nitrogen application had highly significant effects on nitrogen uptake by both plants and fruits (*P* < 0.01). However, their interaction significantly affected only the nitrogen uptake by the fruits (*P* < 0.05, **Figures 4C, D**). As irrigation and nitrogen application levels increased, nitrogen uptake by plants and fruits first increased and then decreased, with the highest uptake observed at W1 and N2. At the same irrigation level, the average nitrogen uptake by plants and fruits under N2 increased by 112.53%, 24.09%, 15.63%, and 54.24%, 31.32%, and 15.02%, respectively, compared with N0, N1, and N3. At the same nitrogen application level, the average nitrogen uptake by plants and fruits at W1 increased by 16.63%, 29.60%, 14.92%, and 9.06%, 24.31%, 41.41%, respectively, compared with W0, W2, and W3.

#### 3.2.2 Soil NO_3_^−^–N

(1) Distribution of soil NO_3_^−^–N.

**Figure 5:**
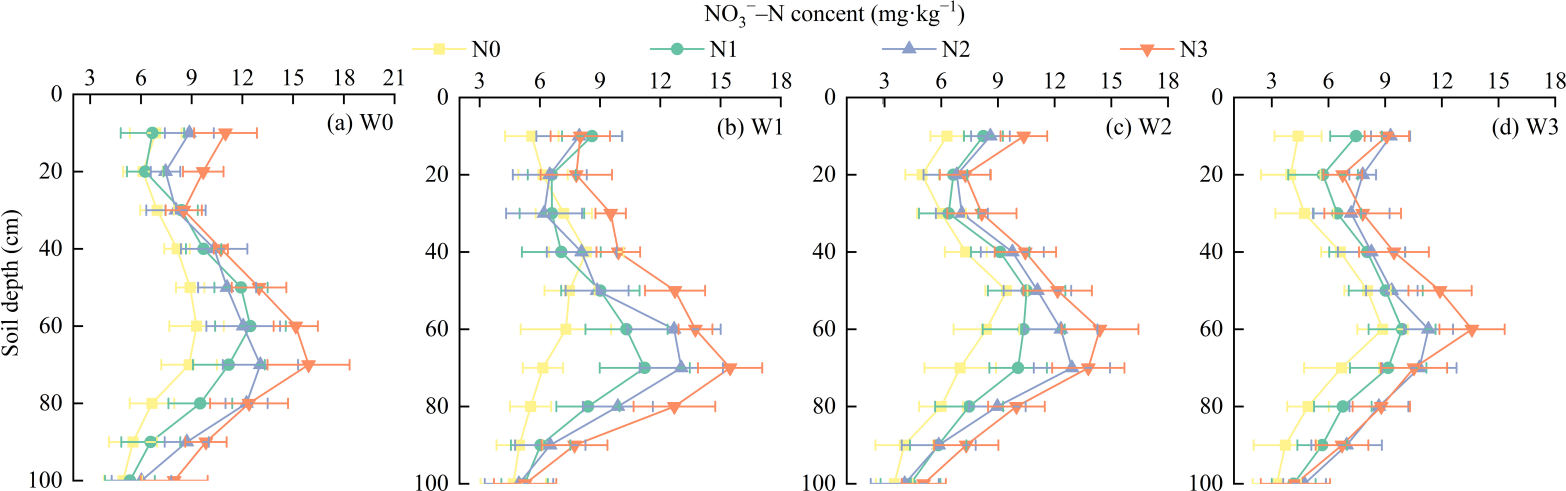
Effects of water and nitrogen regulation on NO3^−^–N distribution in 0–100 cm soil layer. **(A–D)** W0, W1, W2, and W3 represent different water treatment levels: **(A)** W0 (45%-65% field capacity), **(B)** W1 (55%-70% field capacity), **(C)** W2 (65%-80% field capacity), and **(D)** W3 (75%-90% field capacity). N0, N1, N2, and N3 represent different nitrogen treatment levels: N0 (0 kg/ha), N1 (80 kg/ha), N2 (160 kg/ha), and N3 (240 kg/ha). The colors in the line plots indicate the nitrogen levels, where yellow represents N0, green represents N1, blue represents N2, and red represents N3.

The NO_3_^−^–N content in the 0–100 cm soil layer of the wolfberry farmland exhibited a pattern of initially decreased, then increased, and finally decreased again with increasing soil depth (*P* < 0.01, **Figure 5**). The average NO_3_^−^–N content in the 0–100 cm soil layer (5.53–11.42 mg·kg^−1^) generally increased with higher nitrogen application rates, except for N2, and varying irrigation levels. At the same irrigation level, the NO_3_^−^–N content in the 70–90 cm soil layer increased with nitrogen application in the following order: N3 > N2 > N1 > N0. Compared with N1, N2, and N3, the NO_3_^−^–N content in N0 was, on average, 1.46–3.24 mg·kg^−1^, 2.44–5.29 mg·kg^−1^, and 3.33–6.75 mg·kg^−1^ lower, respectively. The NO_3_^−^–N content in the 0–70 cm and 90–100 cm soil layers demonstrated a fluctuating increase in response to increasing nitrogen application. At the same nitrogen application level, the NO_3_^−^–N content in the 0–80 cm soil layer exhibited a fluctuating decrease as irrigation levels increased, whereas the NO_3_^−^–N content in the 80–100 cm soil layer increased with increasing irrigation. Specifically, W0 had, on average, 1.04–1.35 mg·kg^−1^,1.81–1.89 mg·kg^−1^, and 1.89–1.96 mg·kg^−1^ higher NO_3_^−^–N content than W1, W2, and W3, respectively.

(2) Soil NO_3_^−^–N residual.

**Figure 6:**
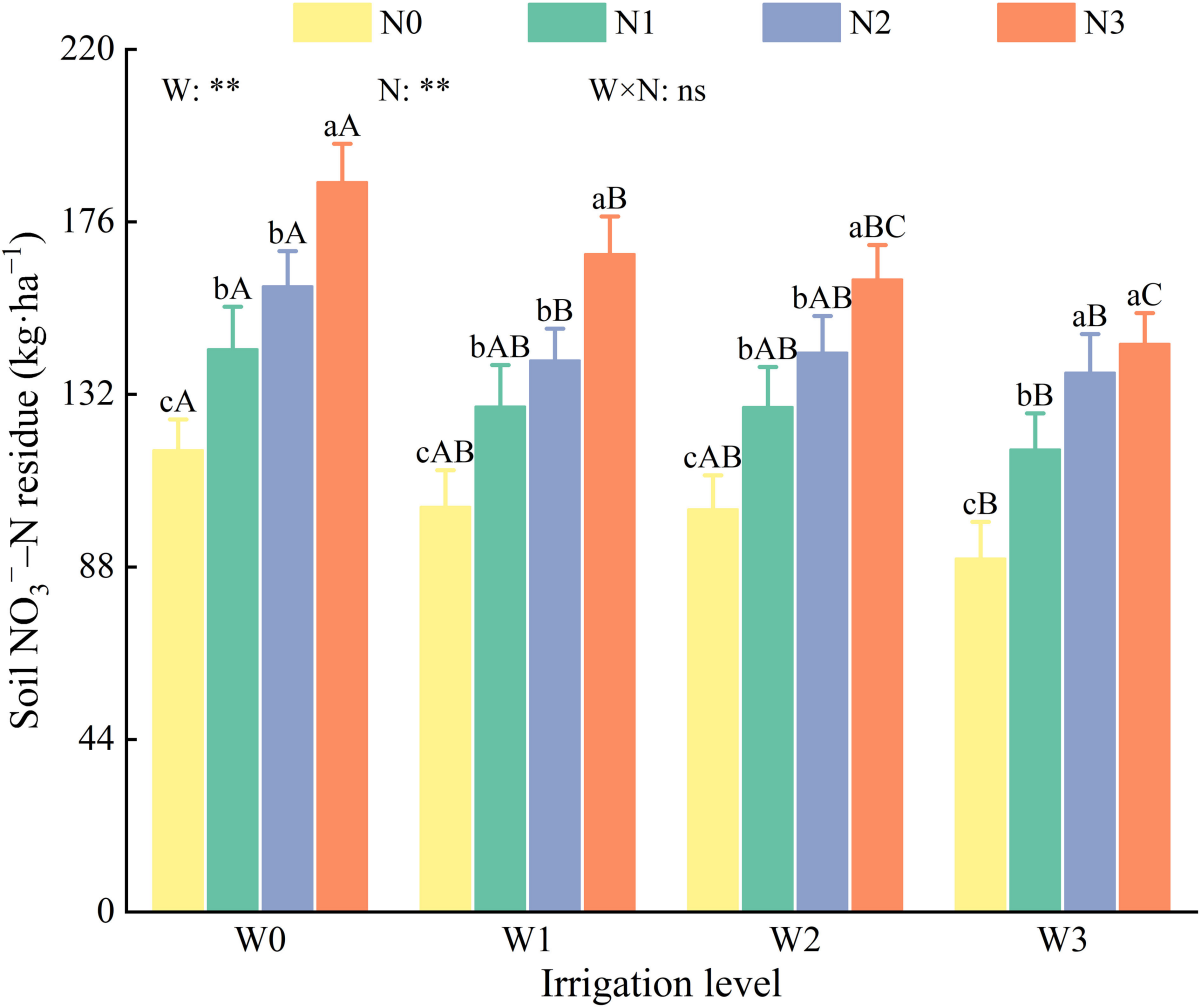
Effects of water and nitrogen regulation on NO_3_^−^–N residue in soil. Different lowercase letters indicate the difference between different nitrogen application levels under the same irrigation management, and different capital letters indicate the difference between different irrigation management types under the same nitrogen application level (*P* < 0.05). W and N refer to irrigation and nitrogen application levels, respectively; N × W refers to interaction effect between the two. ** indicates an extremely significant difference (*P* < 0.01); ns indicates no significant difference (*P* > 0.05).

Irrigation and nitrogen application had highly significant effects (*P* < 0.01) on soil NO_3_^−^–N residuals; however, their interaction did not have a significant effect (*P* > 0.05, **Figure 6**). At the same irrigation level, residual soil NO_3_^−^–N increased with higher nitrogen application rates. Specifically, N0 exhibited significantly lower residuals compared with N1, N2, and N3 by 25.60–27.85 kg·ha^−1^, 37.35–47.45 kg·ha^−1^, and 54.81–68.40 kg·ha^−1^, respectively. Soil NO_3_^−^–N residuals for N0, N1, and N3 increased with increasing irrigation levels. In contrast, for N2, the residuals followed the order of W0 > W2 > W1 > W3 as the irrigation levels increased.

#### 3.2.3 Soil N_2_O emissions

(1) N_2_O emission flux.

**Figure 7:**
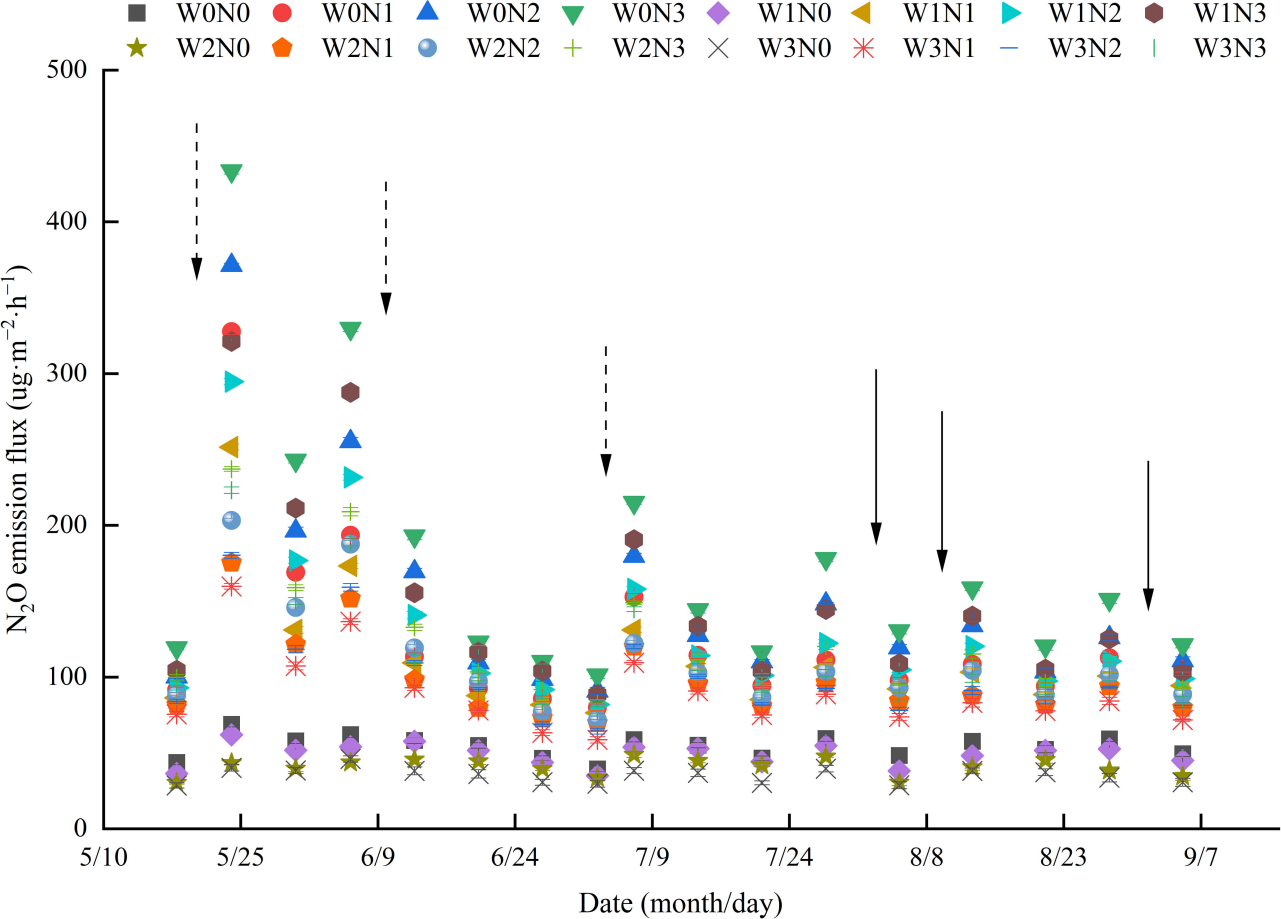
Effects of water and nitrogen regulation on soil N_2_O emission. Note: The dotted arrows in the figure represent nitrogen application by irrigation water, and the solid arrows represent irrigation water.

The soil N_2_O emission flux throughout the entire growth period of wolfberry ranged from 28.50–433.41 μg·m^−2^·h^−1^. A temporary peak in N_2_O emission flux was observed after irrigation and nitrogen application, which subsequently decreased over time. The peak emission flux following irrigation combined with nitrogen application was significantly higher than that observed after irrigation alone (**Figure 7**). At the same irrigation level, soil N_2_O emission flux increased with higher nitrogen application rates. Specifically, the N_2_O emission fluxes in N0, N1, and N2 were reduced by 50.93%–84.13%, 8.77%–41.35%, and 2.95%–22.58%, respectively, compared with N3. At the same nitrogen application level, soil N_2_O emission flux increased with increasing irrigation. The reductions observed were 0.79% to 25.88% for W1, 11.24%–49.37%, for W2, and 17.34%–60.40% for W3. The maximum average soil N_2_O emission flux was observed in the W0N3 treatment (175.71 μg·m^−2^·h^−1^), whereas the minimum was recorded in the W3N0 treatment (34.76 μg·m^−2^·h^−1^).

(2) Total N_2_O emissions.

**Figure 8:**
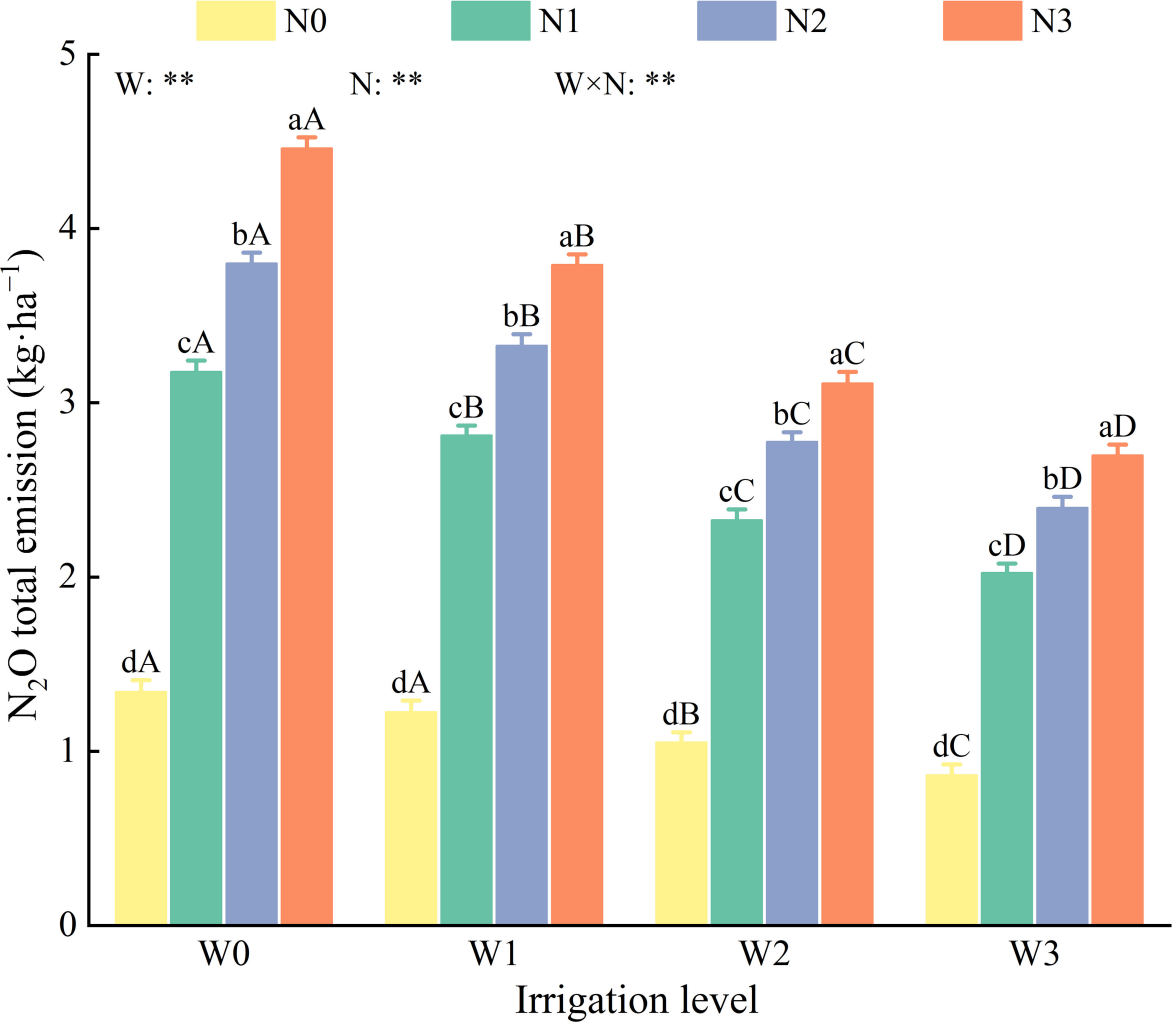
Effects of water and nitrogen regulation on total N_2_O emission from soil. Different lowercase letters indicate the difference between different nitrogen application levels under the same irrigation management, and different capital letters indicate the difference between different irrigation management types under the same nitrogen application level (*P* < 0.05). W and N refer to irrigation and nitrogen application levels, respectively; N × W refers to interaction effect between the two. ** indicates an extremely significant difference (*P* < 0.01).

Water, nitrogen and their interactions had highly significant effects (*P* < 0.01) on the total soil N_2_O emissions (**Figure 8**). Overall, the total soil N_2_O emissions under various water and nitrogen treatments ranged from 0.86–4.46 kg·ha^−1^, demonstrating an increasing trend with higher levels of irrigation and nitrogen application. Compared with N0, the total N_2_O emissions in N1, N2, and N3 were significantly higher by 121.40%–137.11%, 164.12%–183.52%, and 196.11%–232.85%, respectively. Compared with W0, the total N_2_O emissions in W1, W2, and W3 were significantly reduced by 8.60%–15.00%, 21.58%–130.24%, and 35.63%–39.49%, respectively. Among all treatments, the W3N0 treatment exhibited the lowest total N_2_O emissions at 0.86 kg·ha^−1^, which was 17.91%–80.66% lower than those of the other treatments.

#### 3.2.4 Nitrogen outputs in farmland

**Figure 9:**
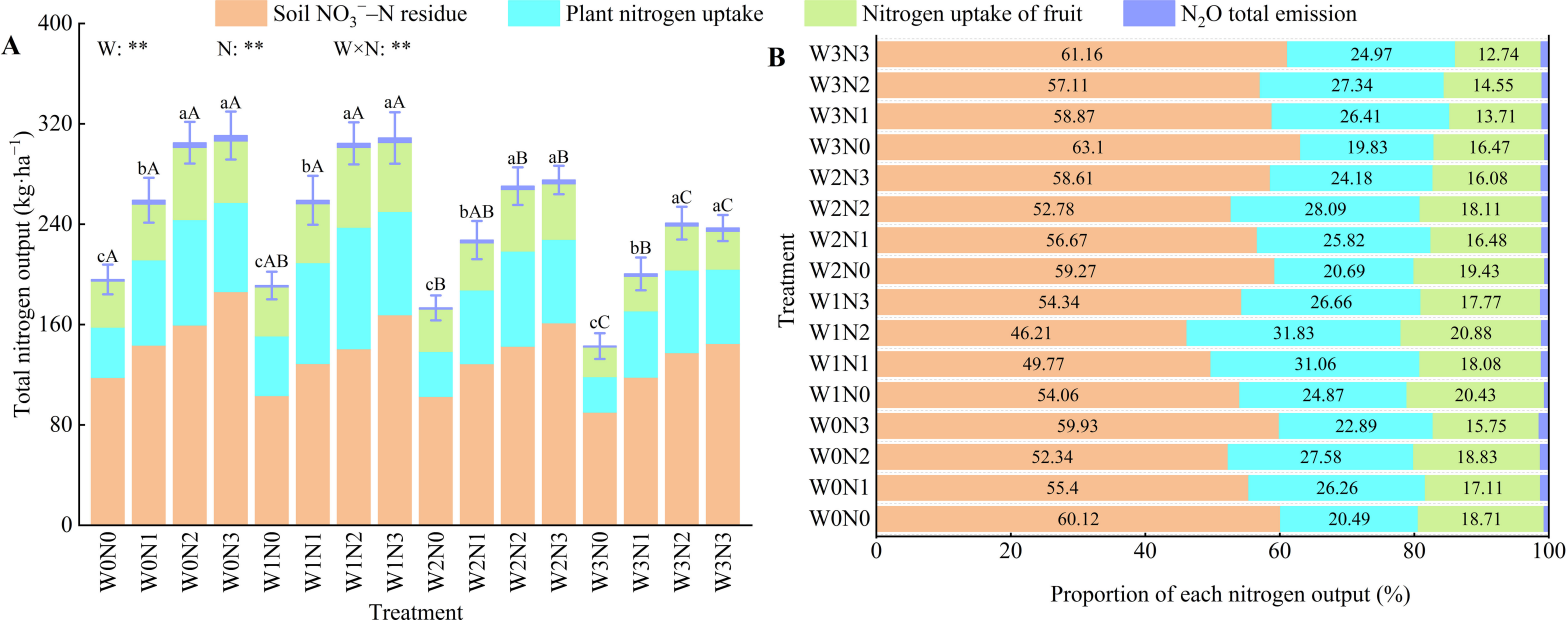
Effects of water and nitrogen regulation on nitrogen output during the growth period of wolfberry. Different lowercase letters indicate the difference between different nitrogen application levels under the same irrigation management, and different capital letters indicate the difference between different irrigation management types under the same nitrogen application level (*P* < 0.05). **(A, B)** represents total nitrogen output and proportion of each nitrogen output, respectively. W and N refer to irrigation and nitrogen application levels, respectively; N × W refers to interaction effect between the two. ** indicates an extremely significant difference (*P* < 0.01).

Water, nitrogen and their interactions had highly significant effects (*P* < 0.01) on total nitrogen output (**Figure 9A**). The total nitrogen output in wolfberry farmland exhibited a decreasing trend with reduced irrigation and nitrogen application levels, with the W3N1 treatment recording the lowest total nitrogen output at 200.39 kg·ha^−1^. The largest proportion of the total nitrogen output was attributed to residual soil NO_3_^−^–N, followed by nitrogen uptake by plants, nitrogen uptake by fruits, and total N_2_O emissions (**Figure 9B**). These components accounted for 49.76%–81.54%, 9.77%–70.22%, 4.70%–25.40%, and 3.36%–18.14% of the total nitrogen output, respectively.

### 3.3 Nitrogen use efficiency of wolfberry under varying water and nitrogen regulations

**Figure 10:**
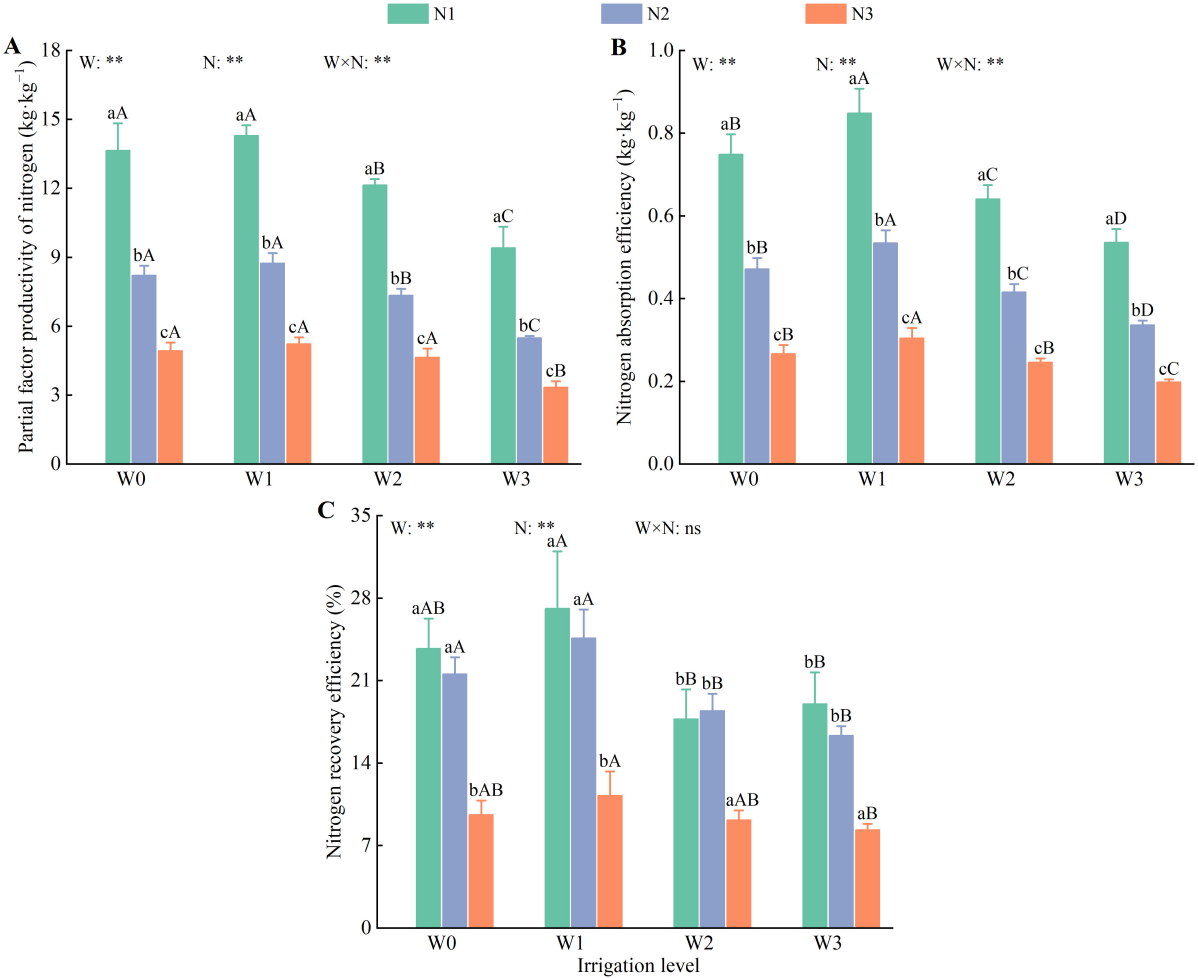
Effects of water and nitrogen regulation on nitrogen use efficiency of wolfberry. Different lowercase letters indicate the difference between different nitrogen application levels under the same irrigation management, and different capital letters indicate the difference between different irrigation management types under the same nitrogen application level (*P* < 0.05). **(A–C)** represents partial factor productivity of nitrogen, nitrogen absorption efficiency and nitrogen recovery efficiency, respectively. W and N refer to irrigation and nitrogen application levels, respectively; N × W refers to interaction effect between the two. ** indicates an extremely significant difference (*P* < 0.01); ns indicates no significant difference (*P* > 0.05).

Irrigation and nitrogen application had highly significant effects (*P* < 0.01) on the partial factor productivity of nitrogen (PFPN), nitrogen absorption efficiency (NAE), and nitrogen recovery efficiency (NRE) in wolfberry. However, the interaction between water and nitrogen had a highly significant effect (*P* < 0.01) on PFPN and NAE (**Figure 10**). The PFPN, NAE, and NRE of wolfberry exhibited a pattern of first increased and then decreased with increasing irrigation (except for W2) and decreased with increasing nitrogen application (except for N1). Compared with W0, W2, and W3, W1 exhibited lower values for PFPN, NAE, and NRE (except for N1) by 4.47%–5.98%, 11.11%–15.95%, and 61.72%–64.40%; 11.72%–12.50%, 19.22%–24.47%, and 34.87%–37.11%; and 12.38%–14.53%, 18.40%–25.06%, and 25.86%–33.57%, respectively. The PFPN, NAE, and NRE values for N2 and N3 (except for W2) were lower than those for N1 by 38.82%–41.67% and 61.72%–64.40%; 35.06%–37.24% and 61.58%–64.40%; and 9.06%–14.08% and 56.22%–59.50%, respectively.

### 3.4 Soil nitrogen balance under varying water and nitrogen regulation

#### 3.4.1 Soil nitrogen balance

**Figure 11:**
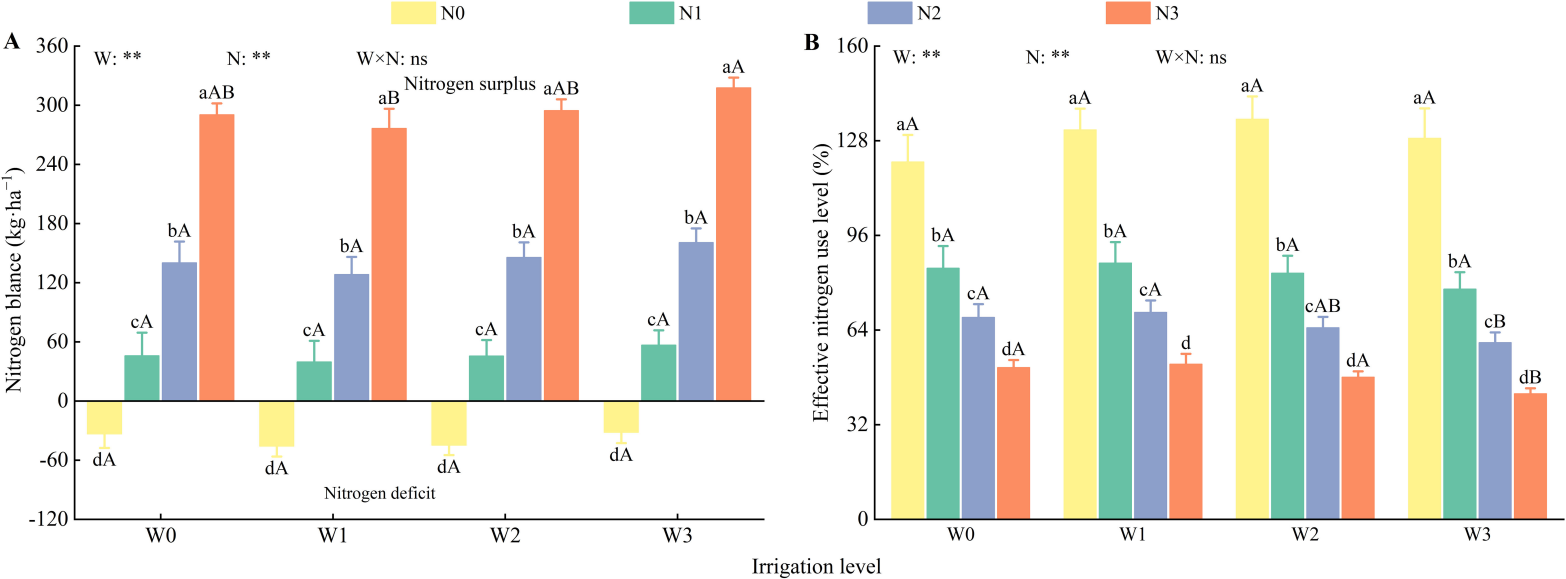
Effects of water and nitrogen regulation on soil nitrogen balance. Different lowercase letters indicate the difference between different nitrogen application levels under the same irrigation management, and different capital letters indicate the difference between different irrigation management types under the same nitrogen application level (*P* < 0.05). **(A, B)** represents nitrogen balance and effective nitrogen use level, respectively. W and N refer to irrigation and nitrogen application levels, respectively; N × W refers to interaction effect between the two. ** indicates an extremely significant difference (*P* < 0.01); ns indicates no significant difference (*P* > 0.05).

Irrigation and nitrogen application had highly significant effects (*P* < 0.01) on soil nitrogen balance (**Figure 11A**). During the wolfberry growth period, all nitrogen application treatments resulted in excess soil nitrogen, whereas all non-nitrogen treatments led to a deficit in soil nitrogen. The excess of soil nitrogen for N1, N2, and N3 was 39.63–56.60 kg·ha^−1^,128.33–160.69 kg·ha^−1^, and 276.36–317.63 kg·ha^−1^, respectively. In contrast, the soil nitrogen deficit for N0 ranged from 31.70– 45.68 kg·ha^−1^. The W1N1 treatment achieved a balanced soil nitrogen state, with a deficit of only 39.63 kg·ha^−1^, which represents 26.42% of the total nitrogen applied. Additionally, nitrogen uptake by plants and fruits under the W1N1 treatment was 16.96% and 26.30% lower, respectively, than that under the W1N2 treatment.

#### 3.4.2 Nitrogen use efficiency in soil

Irrigation and nitrogen application had highly significant effects (*P* < 0.01) on nitrogen use efficiency in the soil (**Figure 11B**). At the same irrigation level, nitrogen use efficiency decreased as nitrogen application increased in the order N0 > N1 > N2 > N3. The nitrogen use efficiency of N1 was 23.87%–30.25% and 65.16%–83.35% higher than that of N2 and N3, respectively. At the same nitrogen application level (except for N0), nitrogen use efficiency decreased with increasing irrigation, following the order W1 > W0 > W2 > W3. Notably, W0 exhibited a reduction of 2.02%–2.42% compared with W1. Among all treatments, W1N1 exhibited the highest nitrogen use efficiency (86.94%), whereas W3N3 demonstrated the lowest efficiency at 42.45%.

## 4 Discussion

### 4.1 Effects of water and nitrogen regulation on wolfberry growth and nitrogen uptake

Water and nitrogen are the primary limiting factors for crop growth in arid and semi-arid regions. Moderately increasing irrigation and nitrogen application can enhance photosynthesis and promote nutrient absorption in crops (Ma et al., 2023; Luo et al., 2020; Liu et al., 2018). This study found that the dry matter of wolfberry plants initially increased and then decreased with increasing irrigation and nitrogen application, following the order W1 > W0 > W2 > W3 and N2 > N3 > N1 > N0. The maximum dry matter was observed under the W1N2 treatment. This finding is consistent with that of a study conducted by Wang et al. (2011b), who concluded that coupling 30 mm of irrigation with 105 kg·ha^−1^ of nitrogen significantly increased the dry matter yield of flue-cured tobacco. Cui and Fang (2016) also found that both water and nitrogen significantly affected the dry matter of flax organs, with the effect of water being greater than that of nitrogen. However, this study suggests that nitrogen has a more significant effect on the dry matter of wolfberry organs than water. This difference may be attributed to the distinct response mechanisms of the various crops to water and nitrogen. Crop yield is closely related to soil moisture levels and nutrient availability (Jiang et al., 2022). Appropriate management of water and nitrogen supply can create a synergistic effect, in which water regulates fertilizer application and fertilizer enhances water utilization, thereby significantly improving crop yield. However, this synergistic effect has a distinct threshold effect on crop growth. Below this threshold, moderate increases in water and nitrogen input can significantly enhance crop growth. However, above this threshold, an increased input may result in reduced yield (Yue et al., 2015). This study supports this pattern, as the fruit yield of wolfberry followed the order W1 > W0 > W2 > W3 and N2 > N3 > N1 > N0.

Soil moisture directly influences nitrogen uptake and utilization in plants, with higher moisture levels significantly enhancing nitrogen absorption. However, this study found that nitrogen uptake by wolfberry plants and fruits followed the pattern of W1 > W0 > W2 > W3 with increasing irrigation, which is consistent with the findings of Tang et al. (2023b) on nitrogen uptake by crested wheatgrass in Zhangye, Gansu. Nitrogen contributes to 40%–50% of crop yield and is a major limiting factor in crop productivity. Proper nitrogen application promotes crop yield while enhancing nitrogen uptake and utilization efficiency. Zhang et al. (2013) observed that the total aboveground nitrogen uptake by maize increased with nitrogen application up to 260 kg·ha^−1^ and then decreased when nitrogen application exceeded 260 kg·ha^−1^. Similarly, this study found that nitrogen uptake by wolfberry plants and fruits followed the order N2 > N3 > N1 > N0 with increasing nitrogen application. This indicates that the uptake of nitrogen by crops is not directly proportional to the amount of nitrogen applied.

### 4.2 Effects of water and nitrogen regulation on soil nitrogen loss in wolfberry farmland

Soil NO_3_^−^–N, a readily absorbable form of inorganic nitrogen for plants, serves as a crucial indicator for evaluating the soil nitrogen supply capacity and the effects of nitrogen fertilization (Hou et al., 2018). It is significantly influenced by irrigation practices and nitrogen levels. The amount of irrigation directly affected the leaching process of soil NO_3_^−^–N. When more nitrogen fertilizer is applied than required by crops and soil microorganisms, excess nitrogen leads to an increase in residual NO_3_/> ^−^–N in the soil. This study found that the NO_3_^−^–N content in the 0–100 cm soil layer initially decreased, then increased, and finally decreased with increasing soil depth. The NO_3_^−^–N content in the 70–90 cm soil layer was significantly higher than that in the 0–70 cm soil layer. The average NO_3_^−^–N content in the 0–100 cm soil layer increased with higher irrigation and nitrogen levels, except for N2. This result is consistent with the findings of Zhang et al. (2024) in the Guanzhong region of Shaanxi, where the NO_3_^−^–N content in the deep soil layer (60–180 cm) was higher than that in the shallow layer. This phenomenon may occur because under repeated excessive irrigation, excess nitrogen that is not absorbed by crops leaches through soil pores and accumulates in specific soil layers. Previous studies have shown that the cumulative amount of NO_3_> ^−^–N in the 0–100 cm soil layer after wheat harvest is positively correlated with nitrogen application, with NO_3_^−^–N accumulation in nitrogen-treated plots being 26.9–162.21 kg·ha^−1^ higher than in non-nitrogen-treated plots (Chen et al., 2015). When nitrogen application exceeded 225 kg·ha^−1^, the NO_3_^−^–N content in the soil profile significantly increased, with greater accumulation observed in the deeper soil layers than in the shallow layers (Wang et al., 2011a). The findings of this study indicate that the accumulation of soil NO_3_^−^–N increases with higher nitrogen application, which is consistent with the results obtained.

N_2_O, the third most significant greenhouse gas after CH_4_ and CO_2_, has a global warming potential that is nine times greater than that of CH_4_ (Zou et al., 2003) and 298 times greater than that of CO_2_ (Jeffry et al., 2021). Agricultural activities are the primary source of soil N_2_O emissions, contributing approximately 78% of global anthropogenic emissions (Smith et al., 2008). Irrigation and nitrogen application are important agricultural management practices that affect soil N_2_O emissions (Xu et al., 2024), primarily by modifying the soil environment and affecting the nitrification and denitrification processes carried out by soil microorganisms (Hu et al., 2013). Studies have shown that irrigation primarily affects factors such as soil redox potential, pore distribution, and aeration, whereas fertilization mainly influences the concentration of substrates required for nitrification and denitrification, thereby affecting the pathways of soil N_2_O emissions (Xie et al., 2011). This study found that the N_2_O emission flux during the entire growth period of wolfberry ranged from 28.50 to 433.41 μg·m^−2^·h^−1^, with six peaks in N_2_O emissions occurring after irrigation and nitrogen application, or irrigation alone. Similarly, Wang et al. (1995) observed that the peaks in N_2_O emissions for summer maize in the North China Plain primarily occurred after irrigation, fertilization, or rainfall. This is likely because irrigation and fertilization enhance soil microbial abundance and enzyme activity (Li et al., 2019), which in turn accelerates the mineralization of organic matter and increases soil nitrogen content. Additionally, consistent with the findings of Du et al. (2018), this study found that the average N_2_O emission flux was 10.85%–63.16% higher under full irrigation than under deficit irrigation. This may be because increased soil moisture reduces soil porosity and O_2_ diffusion capacity, thereby enhancing denitrification. Other studies have demonstrated that the addition of exogenous nitrogen increases the concentration of substrates for nitrification and denitrification, thereby enhancing N_2_O emissions (Stehfest and Bouwman, 2019). This study found that total N_2_O emissions were significantly influenced by nitrogen application, with N1, N2, and N3 showing increases of 1.16–3.12 kg·ha^−1^ in total N_2_O emissions compared with N0, further demonstrating the promoting effect of exogenous nitrogen on N_2_O emissions (Zheng et al., 2022). Similar to the findings of Zheng et al. (2021) in wheat fields in Northwest China, this study also identified a significant positive correlation between total N_2_O emissions and nitrogen application. Therefore, investigating the optimal levels of irrigation and nitrogen application is the most fundamental and effective method for reducing soil N_2_O emissions.

### 4.3 Effects of water and nitrogen regulation on farmland nitrogen balance

The soil nitrogen balance represents the relationship between nitrogen inputs and outputs within the soil, primarily indicating the status of nitrogen sources and sinks. Research indicates that an imbalance in soil nitrogen can lead to either excess or deficit. A high nitrogen surplus not only signifies nitrogen waste but also increases the risk of nitrogen loss. Conversely, nitrogen deficit can reduce soil fertility and weaken plant metabolic activities. This study found that soil nitrogen was in excess in the N1, N2, and N3 treatments, with N1 exhibiting the lowest surplus, which decreased by 64.77% to 69.12% and 82.18% to 85.66% compared with N2 and N3, respectively. The likely reason for this result is that the nitrogen application rate in N3 (450 kg·ha^−1^) significantly exceeded the maximum nitrogen demand of wolfberry, resulting in excessive nitrogen residue in the soil. Additionally, the study observed that in the absence of nitrogen application, the soil nitrogen was in a deficit state, indicating that the soil in the Yellow River irrigation district of Gansu is infertile and requires exogenous nitrogen input to meet the normal growth requirements of wolfberry. Moreover, compared with N2, the N1 treatment, which involved a 50% reduction in nitrogen application, led to only a 14.92%–16.81% decrease in total nitrogen output. This finding is consistent with research conducted by Abera et al. (2018) who indicated that a 50% reduction in nitrogen application led to only a 7%–17% decrease in total nitrogen output. In practical agricultural production, achieving a perfect balance between soil nitrogen input and output is challenging. However, it is possible to maintain a low surplus or deficit of nitrogen by minimizing nitrogen loss without adversely affecting crop growth. This study found that the W1N1 treatment brought soil nitrogen levels closer to a balanced state, with nitrogen uptake by plants and fruits being 16.96% and 26.30% lower, respectively, than that of the W1N2 treatment. This indicates that effective management of water and nitrogen can stabilize soil nitrogen inputs while minimizing nitrogen loss, thereby maintaining soil nitrogen balance. Therefore, it is essential to optimize water and nitrogen management practices for farmlands based on local conditions to achieve high-quality wolfberry production and maintain a balanced soil nitrogen level.

## 5 Conclusions

The total nitrogen input in the wolfberry farmland significantly decreased because of reduced irrigation and nitrogen application. Soil NO_3_> ^−^–N residual accounted for the largest proportion of the total nitrogen output, followed by nitrogen uptake by plants, nitrogen uptake by fruits, and total N_2_O emissions, contributing 49.76%–81.54%, 9.77%–70.22%, 4.70%–25.40%, and 3.36%–18.14% of the total nitrogen output, respectively. W1N1 treatment brought soil nitrogen levels closer to a balanced state. The PFPN (14.29 kg·kg^−1^), NAE (0.85 kg·kg^−1^), and NRE (27.14%) all reached their maximum values under the W1N1 treatment. Considering wolfberry production, nitrogen pollution, and soil nitrogen balance, a combination of 392.40 mm of irrigation and 150 kg·ha^−1^ of nitrogen application represented the optimal model for water and nitrogen regulation in wolfberry cultivation within the Yellow River irrigation district of Gansu Province, China, as well as in other similar arid regions. At the same time, in order to further strengthen the applicability of the research results, the gradient of water and nitrogen application will be narrowed based on the appropriate water and nitrogen threshold in the course of follow-up research, so as to obtain a more accurate water and nitrogen control strategy for water and nitrogen saving, production and efficiency improvement in wolfberry production.

## Data Availability

The original contributions presented in the study are included in the article/supplementary material. Further inquiries can be directed to the corresponding authors.
